# Supplementary material for: Frequency of Use of Added Sugar, Salt, and Fat in Infant Foods up to 10 Months in the Nationwide ELFE Cohort Study: Associated Infant Feeding and Caregiving Practices
Source: Nutrients. 2019 Mar 29;11(4):733. doi: 10.3390/nu11040733 (PMC6520717; doi:10.3390/nu11040733)
Supplement: Supplementary file 1 [file nutrients-11-00733-s001.pdf]

Table 1. Multivariate analyses of associations between the SU of added sugar, salt and fat from 3 to 10 months of age, and familial health and socioeconomic characteristics (N=10 159).

	Sugar		Salt		Fat	
	**β (95% CI)**	**p-value***	**β (95% CI)**	**p-value***	**β (95% CI)**	**p-value***
Infant characteristics						
**Birth rank**		**0.001**		**<0.0001**		**0.001**
First	ref		ref		ref	
Second	0.01 (-0.01;0.02)		0.05 (0.03;0.07)		-0.02 (-0.05;0.02)	
≥Third	0.04 (0.02;0.07)		0.09 (0.06;0.11)		-0.09 (-0.13;-0.04)	
Maternal characteristics						
**Prepregnancy BMI (kg/m²)**		0.62		0.75		0.27
<18.5	0.001 (-0.03;0.03)		0.01 (-0.03;0.03)		0.02 (-0.03;0.08)	
18.5-24.9	Ref		Ref		Ref	
25-29.9	0.003 (-0.02;0.02)		0.01 (-0.01;0.03)		-0.02 (-0.06;0.02)	
≥30	-0.01 (-0.04;0.01)		-0.001 (-0.03;0.03)		-0.04 (-0.09;0.01)	
**Age at delivery (years)**		**0.046**		**0.002**		**0.02**
< 25	0.03 (0.001;0.06)		0.06 (0.03;0.09)		-0.08 (-0.13;-0.02)	
25-29	0.01 (-0.01;0.02)		0.02 (0;0.04)		-0.03 (-0.06;0.01)	
30-34	Ref		Ref		Ref	
≥ 35	0.02 (0.002;0.04)		0.01 (-0.01;0.03)		0.02 (-0.02;0.05)	
**Education level (years)**		0.07		**0.03**		**0.02**
≤9	0.03 (0.001;0.05)		0.02 (-0.01;0.05)		-0.07 (-0.12;-0.03)	
12	-0.01 (-0.03;0.02)		0.02 (-0.004;0.04)		-0.05 (-0.09;-0.01)	
14	0.01 (-0.01;0.03)		0.03 (0.01;0.05)		-0.02 (-0.06;0.01)	
≥15	ref		ref		ref	
Paternal characteristics						
**Age difference with the mother**		0.19		**0.01**		0.18
Younger father	0.02 (-0.003;0.04)		0.02 (0.002;0.05)		-0.03 (-0.07;0.01)	
Father 0-1 years older	Ref		*Ref*		*Ref*	
Father 2-3 years older	0.01 (-0.01;0.03)		0.02 (0.004;0.05)		0.01 (-0.03;0.05)	
Father 4-7 years older	0.02 (-0.01;0.04)		0.02 (0.001;0.04)		0.02 (-0.02;0.05)	
Father at least 8 years older	0.03 (0.01;0.06)		0.04 (0.02;0.07)		0.03 (-0.02;0.08)	
**Education level (years)**		0.70		0.76		**<0.0001**
≤9	-0.002 (-0.02;0.02)		-0.01 (-0.03;0.02)		-0.08 (-0.12;-0.04)	
12	-0.01 (-0.03;0.01)		-0.01 (-0.03;0.01)		-0.08 (-0.12;-0.04)	
14	-0.01 (-0.03;0.01)		-0.001 (-0.02;0.02)		-0.02 (-0.06;0.02)	
≥15	ref		ref		ref	
Household characteristics						
**Parental country of birth**		0.74		**<0.0001**		**0.01**
Both born in France	Ref		ref		ref	
Both born not in France	0.02 (-0.02;0.06)		0.19 (0.14;0.23)		0.11 (0.02;0.19)	
Mother in France, father not in France	0.02 (-0.02;0.05)		0.09 (0.06;0.12)		0.08 (0.02;0.14)	
Father in France, mother not in France	0.01 (-0.02;0.04)		0.09 (0.06;0.13)		0.04 (-0.02;0.11)	
Study design characteristics						
**Maternity unit size** (number of deliveries/year)		0.36		**0.03**		0.75
145-699	0.04 (-0.01;0.09)		0.003 (-0.05;0.06)		-0.01 (-0.11;0.09)	
700-1,009	Ref		Ref		Ref	
1,010-1,418	0.02 (-0.02;0.05)		-0.03 (-0.06;0.01)		0.02 (-0.04;0.08)	
1,422-2,187	0.01 (-0.02;0.03)		-0.04 (-0.07;-0.01)		0.03 (-0.03;0.08)	
2,197-5,215	0.01 (-0.01;0.04)		-0.04 (-0.07;-0.01)		0.03 (-0.02;0.08)	
**Season of inclusion**		0.96		0.93		**0.01**
From April 1 to April 4	0.01 (-0.02;0.03)		0.001 (-0.02;0.02)		-0.05 (-0.09;-0.01)	
From June 27 to July 4	0.004 (-0.01;0.02)		-0.01 (-0.03;0.01)		-0.06 (-0.09;-0.02)	
From September 27 to October 4	0.001 (-0.02;0.02)		0 (-0.02;0.02)		-0.02 (-0.05;0.02)	

From November 28 to December 5	Ref		Ref		Ref	
**Residential region**		**<0.0001**		**<0.0001**		**<0.0001**
East-Parisian basin	-0.06 (-0.09;-0.03)		-0.06 (-0.10;-0.02)		-0.26 (-0.32;-0.19)	
West-Parisian basin	-0.09 (-0.12;-0.05)		-0.10 (-0.14;-0.07)		-0.24 (-0.30;-0.17)	
Parisian region	-0.05 (-0.08;-0.02)		-0.06 (-0.09;-0.02)		-0.22 (-0.27;-0.16)	
East	-0.05 (-0.08;-0.02)		-0.08 (-0.12;-0.05)		-0.25 (-0.31;-10.2)	
Mediterranean region	-0.01 (-0.04;0.02)		-0.02 (-0.06;0.01)		0.13 (0.07;0.19)	
North	-0.05 (-0.09;-0.02)		-0.10 (-0.13;-0.06)		-0.22 (-0.29;-0.16)	
West	-0.04 (-0.07;-0.01)		-0.11 (-0.14;-0.08)		-0.23 (-0.29;-0.18)	
South-east	-0.01 (-0.04;0.02)		-0.05 (-0.08;-0.02)		-0.14 (-0.20;-0.08)	
South-west	Ref		Ref		Ref	

In bold: significant variable ($p<0.05$). The SU of an ingredient was calculated as the mean frequency of use from the complementary feeding introduction to 10 months. Each month, the parents ranked the use as 0: never, 1: sometimes, 2: often, 3: always or almost always. Consequently, for a given infant, the potential minimal score could be zero, which means that the infant never received the ingredient from the CFI age to 10 months. The potential maximal score could be 3, which means that the infant always received the considered ingredient at every month of the follow-up.

Linear regressions also adjusted for the feeding and caregiving practices presented in the Table 2: Breastfeeding duration, age at complementary feeding introduction, information sources about infant caregiving: family, media, health professionals, maternal personal experience, maternal concern about her child's health and maternal reaction when her child did not eat much at two months. The r^2 of each model are 0.02, 0.04 and 0.10 for SU of added sugar, salt and fat, respectively. Abbreviations: CI, confidence interval; BMI, body mass index; SU, score of use
